# Home health care and cardiac rehabilitation following major cardiac surgeries in Pakistan

**DOI:** 10.1097/MS9.0000000000000865

**Published:** 2023-06-05

**Authors:** Varsha Kumari, Usha Kumari, Syeda A. Batool, Nikhil R. Daggula, Aarash Khan

**Affiliations:** a Department of Medicine, Dow University of Health Sciences, Karachi; b Holy Family Hospital, Rawalpindi, Pakistan; c Kakatiya Medical College, Warangal, Telangana, India; d Herat University, Herat, Afghanistan

**Keywords:** home care aide, cardiovascular rehabilitation, home care service, cardiopulmonary bypass

## Abstract

Cardiovascular disorders are a leading cause of morbidity and mortality globally, and coronary artery bypass graft surgery is one of the most effective procedures for coronary artery disease. Cardiac rehabilitation (CR) has been shown to offer benefits beyond reducing mortality and morbidity rates, including enhancing patients’ quality of life and reducing healthcare costs. Home-based CR programs offer personalized plans tailored to individual needs and availability and have been shown to be more effective in sustaining improvements than center-based CR programs. However, there are challenges associated with providing home care services in developing countries, including personnel shortages, lack of financing and policies, and limited access to end-of-life or hospice services. The use of multidisciplinary telehealth and telecare homecare programs that make use of web-based technologies to monitor postoperative outcomes in patients undergoing cardiac surgery may provide a solution to some of these challenges. This manuscript emphasizes the potential of home health care and CR in improving postoperative outcomes in Pakistan and identifies some of the challenges and potential solutions associated with providing home care services.

Cardiovascular disorders are a leading cause of morbidity and mortality globally, which place a significant burden on healthcare resources^1^. Around 17 million people worldwide are affected by cardiovascular illnesses, which account for more than 30% of deaths annually. Among them, 40% suffer from coronary artery disease (CAD), with coronary artery bypass graft surgery (CABG) serving as one of the most effective procedures for CAD^2^.

A cross-sectional study conducted in a tertiary care hospital in Peshawar, Pakistan, that involved a total of 1613 patients who underwent CABG over 4 years from June 2017 to 2020 reported a comparably elevated rate of in-hospital mortality, about 3.4% in follow-up assessment with dyslipidemia being the leading risk factor in 71.8% of cases. This is considerably higher than seen in developed nations, particularly China and the United States, where the rates were lower at 1.2 and 1.3%, respectively^3^. Cardiac rehabilitation (CR) is a proven intervention that offers benefits beyond reducing mortality and morbidity rates for adults with ischemic heart disease, heart failure, or cardiac surgery. It also enhances their quality of life, decreases hospital readmissions, and, as a result, reduces healthcare costs^4^. A study conducted on physician assistant home care programs (PAHC) following cardiac surgery discovered a 25% decrease in hospital readmission rates, with infection-related readmissions dropping from 44 to 19%. This highlights the effectiveness of home visits on postoperative outcomes, including early detection and management of wound infections. It minimizes severe complications of sepsis and pneumonia in patients after cardiac surgery and promotes compliance with therapy^5^. However, home-based CR (HBCR) and center-based CR (CBCR) programs are both effective in reducing secondary outcomes and readmissions^4^. CBCR programs face several obstacles, including transportation barriers. However, nowadays, HBCR programs involving telemedicine that offer remote coaching and personalized plans tailored to individual needs and availability can help overcome these challenges. As a result, patients are more likely to fully utilize these programs^4^. An experiment conducted in the Kingdom of Saudi Arabia (KSA) through a randomized control trial demonstrated that HBCR programs are more effective in sustaining improvements^6^. To achieve this objective, a multidisciplinary telehealth and telecare homecare program can be employed. A 15-year study on this program, involving 1635 patients with chronic diseases, found that more patients adhered to rehabilitation protocols and reported high satisfaction levels, as did their caregivers^7^. This potential approach makes use of web-based technologies to monitor postoperative outcomes in patients undergoing cardiac surgery, particularly making it convenient after the coronavirus disease 2019 (COVID-19) pandemic, to limit hospital visits^8^. This study discovered that using remote patient monitoring substantially decreased repeat readmission rates by 6.3%, according to remote monitoring evaluation (RME). Out of a total of 2328 patients, which were monitored after cardiac surgery, 2.8% experienced severe pain, 0.9% encountered hypertensive attacks, and sinus tachycardia was observed in 0.4% of the patients, and these issues could be tackled with the novel remote intervention given the patient’s current health status^8^. However, there are certain pitfalls associated with home care services, and one of the downsides being that the nursing staff and healthcare teams from developing countries carry out more duties in contrast to high-income nations, implying a lack of resources in the developing world. Personnel shortages, a lack of financing and policies, limited access to end-of-life or hospice services, and a lack of community awareness services available are all significant additional barriers to delivering home care^9^.

HBCR is vital for the recovery of patients after major cardiac surgeries. The initial postoperative phase poses significant emotional and physical challenges. In developing nations like Pakistan, cardiac patients not only suffer from the severity of their illness but also the insufficiency of the resources available as there is a lack of proper infrastructure and appropriate equipment in healthcare and routine rehabilitation settings. Studies are being conducted to assess the effectiveness of home health care in improving patients’ overall well-being and quality of life. Similar research carried out in Mumbai, India, whose healthcare sector and infrastructure are quite comparable to ours, reported that implementation of home cares palliative services turned out to be a huge success in the management of symptomatic control, particularly in lower-class or middle-class socioeconomic rehabilitation^10,^ providing substantial evidence to endorse these initiatives in other developing nations. Consequently, we should take note and incorporate these services into our healthcare system to alleviate the burden on an already struggling healthcare system with overcrowded medical units. Home-based care can serve as a potentially easily accessible, cost-effective strategy to provide psychosocial support and ensure improved adherence among cardiac surgery patients, particularly in rural areas where patients who need specialized postoperative care often miss out on critical follow-ups. Additionally, it reduces health-related expenses associated with readmissions in impoverished nations where there is a lack of financial stability which highlights the need for these HBCR program initiatives. It is critical to explore these aspects of cardiac care and effectively train more groups of healthcare professionals, opting for a multidisciplinary approach to ensure optimal care improving patient outcomes and lowering morbidity and mortality rates. Urgent action is required from healthcare providers and policymakers to underscore the vital role of these services, raise public awareness, and implement home-based rehabilitation programs for cardiac surgery patients in Pakistan. However, more studies on a large scale should be done to further assess the role of home health care in improving outcomes postoperatively in cardiac surgery patients.

## Ethical approval

Not applicable.

## Consent

Not applicable.

## Source of funding

None to declare.

## Author contribution

V.K.: idea conceptualization, writing original draft, and study design; U.K.: writing original draft and project administration; S.A.B.: writing original draft, proofreading, and formatting; N.R.D.: project administration, proofreading, formatting, and reviewing; A.K.: editing, project administration, and proofreading.

## Conflicts of interest disclosure

No conflict of interest to disclose.

## Research registration unique identifying number (UIN)

Not applicable.

## Guarantor

Usha Kumari.

## Data availability statement

Not applicable.

## Provenance and peer review

Not commissioned, externally peer-reviewed.
